# Update on HDM Allergy: Principal Changes over the Years

**DOI:** 10.3390/ijms26125660

**Published:** 2025-06-13

**Authors:** Krzysztof Jurkiewicz, Marek Jutel, Sylwia Smolinska

**Affiliations:** Department of Clinical Immunology, Faculty of Medicine, Wroclaw Medical University, Parkowa 34, 51-616 Wroclaw, Poland; krzysztof.jurkiewicz@student.umw.edu.pl (K.J.); marek.jutel@umw.edu.pl (M.J.)

**Keywords:** house dust mite allergy, *Dermatophagoides pteronyssinus*, *Dermatophagoides farinae*, allergen-specific immunotherapy, Der p 1, recombinant allergens, asthma, allergic rhinitis

## Abstract

House dust mites (HDMs) are a major source of indoor allergens, significantly contributing to allergic rhinitis, asthma and atopic dermatitis. This review examines the epidemiology, microbiological classification and pathophysiology of HDM allergy, highlighting key allergens such as Der p 1, Der p 2 and Der p 23. Furthermore, we discuss the pivotal role of allergen-specific immunotherapy (AIT), the only disease-modifying treatment for immunoglobulin (Ig)-E disease. Recent studies have identified predictive biomarkers for allergen-specific immunotherapy (AIT) efficacy, including the specific IgE to total IgE (sIgE/tIgE) ratio and regulatory follicular T cell profiles, supporting a more personalized approach to therapy. Additionally, emerging immunotherapy strategies, such as recombinant allergens and peptide-based formulations, aim to improve safety and clinical outcomes. As HDM allergy prevalence rises globally, further research into optimizing diagnostics and treatment strategies remains crucial for enhancing patient care.

## 1. Introduction

House dust mites—HDMs—are one of the most common sources of indoor allergens and are an important etiological factor in allergic rhinitis, allergic asthma and atopic dermatitis [1,2,3]. It is estimated that allergy to mites affects between 4 and 6% of the world’s population, meaning that up to 500 million people are affected by these allergens [4,5]. The most commonly sensitized mite species are *Dermatophagoides pteronyssinus* and *Dermatophagoides farinae*, which are common in temperate and tropical climates [6,7,8,9].

The diagnosis of allergy to mites includes analysis of clinical history, skin tests (prick tests), provocation tests and determination of allergen-specific IgE antibodies. Despite significant advances in diagnosis, allergen extracts that contain both allergenic and non-allergenic components are still used, which can affect the effectiveness of allergy identification [10,11,12]. To date, 40 groups of mite allergens have been identified and classified, but in clinical practice, extracts used for diagnosis and immunotherapy are most often standardized for only the major *D. pteronyssinus* allergens, such as Der p 1 and Der p 2. As a result, some patients who are allergic to other allergenic components may not be properly diagnosed [13,14].

AIT is the only available treatment capable of modifying the course of allergic disease. It has been shown to be effective in the treatment of both allergic rhinitis and allergic asthma, with the selection of appropriate allergen extracts being crucial. Standard immunotherapy formulations, both subcutaneous (SCIT) and sublingual (SLIT), predominantly contain Der p 1 and Der p 2 but often lack adequate amounts of Der p 23. Although Der p 23 has only relatively recently been identified, it is now well established as a major house dust mite allergen. The absence of this component in many vaccine preparations likely contributes to the reduced effectiveness of allergen immunotherapy (AIT) in patients sensitized to Der p 23. Research is currently underway on the use of recombinant allergens and individually purified protein components to improve the efficacy and safety of immunotherapy [3,7,9,12,13].

Due to the high prevalence of sensitization to mite allergens and their impact on the course of allergic diseases and asthma, further improvements in diagnostic and therapeutic methods are needed. Many questions still remain about the immunological mechanisms, the individual response of patients to immunotherapy and the impact of specific allergens on the course of the disease [15].

This paper aims to discuss the epidemiology of house dust mite allergy, the underlying immunological mechanisms as well as current and future diagnostic and therapeutic strategies, with a focus on specific immunotherapy and its potential modifications.

## 2. Epidemiology

HDM allergy is particularly prevalent among patients with allergic asthma, with as many as 90% of people showing hypersensitivity to HDM allergens. In countries with humid and warm climates, such as Malaysia, the percentage of people allergic to mites is as high as 60–80% of the population [4,5].

Its prevalence varies across age groups and regions. In children, sensitization to HDM allergens often begins early in life. Studies indicate that up to 68.3% of children with HDM-induced allergic rhinitis develop symptoms by the age of 6, with a notable increase in prevalence during preschool and school years. Among children with asthma, the rate of HDM sensitization rises with age: from 53.5% in those aged 0–3 years to 80.2% in those aged 8–12 years. In adults, the prevalence of HDM sensitization remains significant. For instance, in the United States, approximately 20 million people are affected by dust mite allergy. In Europe, sensitization rates vary but can be as high as 45% in certain adult populations. Among the elderly, HDM sensitization persists, though patterns may shift. Research indicates that individuals over 70 years may exhibit increased sensitization to storage mites like *Tyrophagus putrescentiae* compared to *Dermatophagoides pteronyssinus*. This shift could be attributed to changes in environmental exposures and immune system alterations with age [9,16].

Overall, HDM allergy demonstrates a significant burden across all age groups, underscoring the importance of age-specific diagnostic and management strategies. Climate change significantly influences the epidemiology of HDM allergy by altering environmental conditions that affect mite proliferation and allergen exposure. Rising global temperatures and increased humidity levels create favorable conditions for HDM survival and reproduction. HDMs thrive in warm, humid environments, and climate-induced changes such as more frequent extreme weather events (e.g., floods) can lead to increased indoor dampness, further promoting HDM proliferation.

Additionally, climate change may lead to shifts in HDM habitats, expanding their range into previously non-endemic areas, thereby exposing new populations to HDM allergens. Urbanization and energy-efficient building designs, aimed at reducing carbon footprints, often result in reduced ventilation and increased indoor humidity, inadvertently creating ideal environments for HDMs. These environmental changes contribute to higher HDM allergen concentrations indoors, increasing the risk of sensitization and exacerbation of allergic diseases such as asthma and allergic rhinitis [16,17,18].

### 2.1. Geographic Distribution and Factors Affecting Epidemiology

The incidence of mite allergy depends on climatic conditions and housing structure. Mites thrive best in environments with high humidity (above 50%) and temperatures in the range of 18–30 °C. Therefore, their presence is particularly high in tropical regions and in buildings with poor ventilation and high dust accumulation [19,20].

In Europe, there is considerable variation in the incidence of HDM sensitization by region. In countries with warmer climates, such as Spain and Italy, mite allergy is more common than in northern countries such as Norway and Finland, where dry air during the winter season limits the mite population [7,8,21].

In Poland, allergy to mites is widespread, especially among children and adolescents. It is estimated that the prevalence of allergy to *Dermatophagoides pteronyssinus* and *Dermatophagoides farinae* in the pediatric population is about 30–40% [22,23,24]. The increase in the number of cases of HDM allergy in Poland is associated with increasing urbanization, increasing use of air conditioning and limited ventilation of homes during the winter.

### 2.2. Impact of Housing Conditions and Lifestyle

Living conditions have a significant impact on exposure to mite allergens. A study conducted in China, where 189 dust samples from floors and air conditioning filters in homes, schools and hotels were analyzed, found that 34.67% of air conditioning filter samples and 20.18% of floor samples were found to contain dust mites. Significantly, the highest density of mites was found in households (10/g), followed by schools (9/g) and the least in hotels (4/g), suggesting that hygiene levels and cleaning frequency play a key role in reducing allergen exposure [25].

## 3. Microbiological Classification

House dust mites are microscopic arachnids belonging to the cluster Arachnida, order Acariformes. Within this order, several families are distinguished, the most important of which in the context of allergology are Pyroglyphidae, Glycyphagidae, Acaridae and Echimyopodidae. The following Table 1 presents detailed classification and characterization of the most common species of house dust mites [6,7,8,9,26,27,28,29,30].

Please note that the above division takes into account the current state of knowledge. Identification studies of further groups of pathogenic mites are constantly being conducted.

## 4. Allergens Relevant to Pathomechanism

To this day, 40 major house dust mite allergens have been identified, which differ in structure, biological function and ability to provoke allergic reactions. Among them, the most clinically relevant are Der p 1, Der p 2 and Der p 23, which are characterized by high immunogenicity and are among the main factors provoking allergic symptoms in people allergic to mites.

Der p 1 is a cysteine proteolytic enzyme that degrades proteins that build the airway epithelial barrier, thereby facilitating the penetration of other allergens and enhancing the immune response. Der p 2, although not an enzyme, shows structural similarity to toll-like receptor 4 (TLR4), leading to the activation of inflammatory pathways in the body. Der p 23, on the other hand, is a relatively newly discovered allergen that plays a key role in stabilizing the mite barrier and at the same time shows high IgE-binding capacity, making it one of the most important sensitizers.

In addition to these three major allergens, there are a number of other mite proteins, such as proteolytic enzymes (Der p 3, Der p 6), lipid-binding proteins (Der p 2, Der p 21), tropomyosins (Der p 10) or chitinases (Der p 15), which can also trigger severe allergic reactions. Each of these allergens can have varying degrees of clinical relevance, and their combination in environmental exposures plays a key role in the development of allergic symptoms, including allergic rhinitis, bronchial asthma and atopic dermatitis.

The identification and detailed understanding of individual mite allergens is important for diagnosis, treatment and the development of effective AIT methods that can significantly reduce the severity of allergic reactions in patients [31,32,33,34,35,36,37,38,39,40,41,42,43,44,45,46,47,48,49,50,51,52,53].

The following summary Table 2 shows each allergen along with its functions and ability to produce specific IgE class antibodies.

## 5. Molecular and Cellular Basis of Allergic Response to House Dust Mite

In recent years, there have been important developments in understanding the molecular basis of allergy to house dust mites, in particular the species *Dermatophagoides pteronyssinus*. Increasing evidence indicates that the host immune response is not just the result of classical activation of Th2 lymphocytes by specific antigens, but it also results from deep-rooted molecular interactions—both at the level of allergen structure and cellular metabolism of immune and epithelial cells. Allergic reactions induced by house dust mites are of a complex immunological and molecular nature, involving both innate and adaptive immunity mechanisms. The main components of these reactions include the activation of immune cells (macrophages, dendritic cells, Th2 lymphocytes), expression of pro-inflammatory cytokines, remodeling of the bronchial epithelium and epigenetic processes and signal transduction dependent on environmental co-factors (e.g., pollution particles).

### 5.1. The Lipid Properties of Allergens and Their Effect on Immunogenicity

One of the important mechanisms modulating the immunogenicity of HDM allergens is their lipophilic properties and their ability to bind lipids and present them in the context of receptors of innate immunity. Allergens such as Der p 2, Der p 5, Der p 7, Der p 13 and Der p 21 show the ability to interact with lipids through hydrophobic pockets and lipid-binding domains. Der p 2 shows structural similarity to the MD-2 protein, a coreceptor for TLR4, which enables it to directly activate this receptor in the respiratory epithelium and induce a Th2 response without the need for lipopolysaccharides (LPS). Der p 13 allergen, on the other hand, selectively binds fatty acids in its inner hydrophobic cavity and activates epithelial cells through TLR2-MyD88 and NF-κB and MAPK-dependent pathways.

These interactions not only increase the immunogenicity of the proteins but also affect their resistance to enzymatic degradation and facilitate their penetration through the mucosal barrier, which may determine the effectiveness of sensitization. In addition, some HDM-associated lipids act as adjuvants, intensifying the immune response and promoting the development of the asthma phenotype [40,54].

### 5.2. Immunometabolic Reprogramming of Myeloid Cells

Novel studies on the immunometabolism of myeloid cells reveal that exposure to HDM leads to strong metabolic reprogramming of macrophages and dendritic cells. As shown, HDM induces a simultaneous increase in both glycolysis and mitochondrial respiration in macrophages (BMDM), alveolar macrophages (BMDAM) and dendritic cells (BMDC). This pattern of activation is not characteristic of classical M1/M2 polarization states, suggesting a unique activation phenotype with high pro-inflammatory potential. The increase in metabolic activity is coupled to the production of cytokines, including TNF-α, and depends on TLR4 activation—both by LPS and HDM components such as β-glucan.

It is noteworthy that it is the combination of β-glucan and LPS (present in HDM) that leads to a particularly strong pro-inflammatory activation, including not only TNF production but also increased reactive oxygen species (ROS) production, altered mitochondrial morphology and intensification of oxidative metabolism. This mechanism appears to be linked to trained immunity, leading to permanent reprogramming of innate immune cell function [55].

### 5.3. TRPC1 Calcium Channels and Airway Remodeling

Another finding that expands the understanding of the molecular basis of HDM allergy is the role of TRPC1 (transient receptor potential canonical 1) channel. In a mouse model, TRPC1 deficiency was shown to significantly reduce airway remodeling symptoms after HDM exposure—reducing mucus production (MUC5AC), collagen deposition and smooth muscle hypertrophy. Mechanistically, TRPC1 mediates Ca^2+^ ion influx by activating the STAT3 pathway, which promotes epithelial–mesenchymal transition (EMT) and mucin expression.

The importance of this pathway is not limited to the remodeling of bronchial architecture. Enhanced TRPC1 activity also increases the inflammatory response by activating NF-κB and promoting the secretion of cytokines such as IL-6 and IL-1β. This implies that TRPC1 may act as a “gateway” for environmental signals leading to severe chronic asthma [55].

### 5.4. Macrophages and Phenotype Polarization in Response to HDM

Macrophages are the first line of defense in response to inhalant allergens. In the context of HDM, their activation proceeds dynamically along the M1-M2 axis, with M1 macrophages producing pro-inflammatory cytokines (TNFα, IL-6, IL-1β) and M2 macrophages producing IL-4 and Retnl cytokine, involved in tissue remodeling and wound healing. The work of Dunbar et al. showed that the expression of the MIF gene promoter polymorphism (CATT7) promotes pro-inflammatory activation of macrophages (M1) in response to HDM and LPS, which can lead to sustained, hyper-reactive inflammation in the airways. In addition, features of “immune training”—a permanent change in the phenotype of macrophages as a result of previous exposure to HDM, which is associated with epigenetic reprogramming (blocked by the methyltransferase inhibitor MTA)—have been observed [56].

### 5.5. Differentiation of Monocytic and Macrophage Responses

Fransen and Leonard’s study using single-cell RNA sequencing showed that macrophage subpopulations (e.g., MCSPP1^+^ and MLCMARCO^+^) differ in their transcriptional response to HDM, particularly in terms of the expression of chemokines such as CXCL5, CXCL8, CCL5 and CCL15. Alternatively activated macrophages (MLCCD206^+^, corresponding to M2) showed the greatest changes in gene expression. The presence of diesel exhaust particles (DEPs) further enhanced the response, suggesting a synergistic effect of allergens and air pollutants in the induction of asthma [57].

### 5.6. Epithelial–Mesenchymal Transition (EMT) and Epithelial Remodeling

Airway remodeling, characteristic of chronic asthma, also involves the epithelial–mesenchymal transition (EMT) process. Yoshie et al. described that HDM and the cytokine TGF-β1 induce EMT through Smad and non-Smad pathways (PI3K/AKT, Wnt/β-catenin), leading to a decrease in E-cadherin expression and an increase in the expression of mesenchymal markers (e.g., αSMA, fibronectin, Snail1). Importantly, chloride channels (such as ClC-2, LRRC8 and TMEM16A) play a role in cell volume regulation and may participate in EMT by altering cell volume and activating morphogenetic signals [58].

### 5.7. Influence of Genetic and Epigenetic Determinants: Disruption of Epithelial Barrier Integrity and Activation of Structural Cells

The presence of polymorphisms (e.g., CATT7 in the MFI gene) affects the “training” of innate immunity and susceptibility to severe asthma. Exposure to HDMs in such individuals leads to the perpetuation of a pro-inflammatory macrophage phenotype, which can exacerbate the chronic nature of the disease. Epigenetic changes (e.g., DNA methylation, chromatin alteration) are important in this mechanism and are increasingly being considered as a therapeutic target.

**Figure 1:**
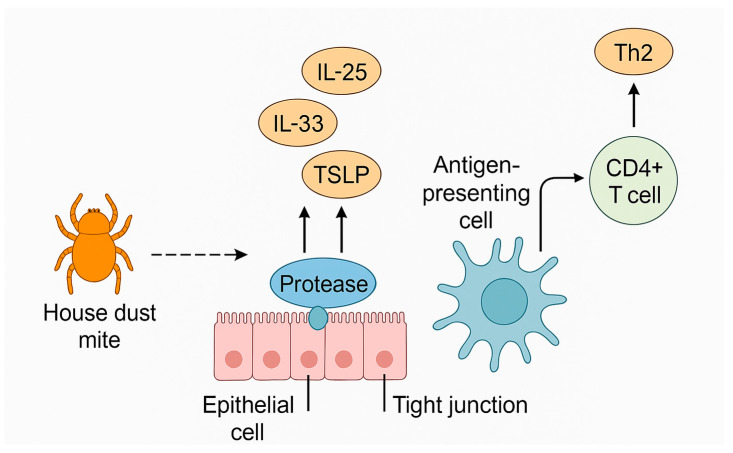
Mechanism of action of protease allergens in damaging tight intercellular junctions and activating Th2-type immune response.

Allergens with protease activity cleave tight junctions (TJs) in proteins such as claudins, occludin or ZO-1, leading to increased permeability of the respiratory epithelium and facilitating allergen penetration into subepithelial tissues. Der p 1 can also degrade local protease inhibitors (e.g., elafin, α1-antitrypsin), impairing host protective mechanisms. Alarmin molecules (IL-33, TSLP, IL-25) released as a result of epithelial damage activate dendritic cells and promote the polarization of the immune response toward Th2 [59]. Mechanism of action of protease allergens is presented on Figure 1.

### 5.8. Involvement of Toll-like Receptors (TLRs) in Modulation of Immune Response

**Figure 2:**
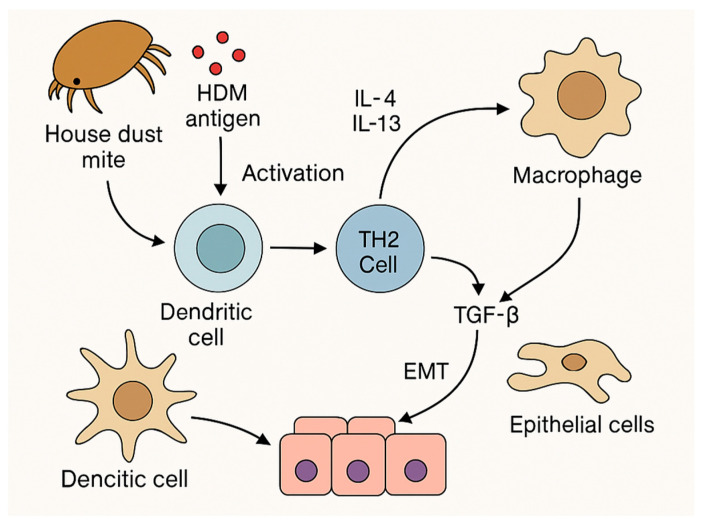
A diagram of Th2 response activation by house dust mite antigens involving dendritic cells, IL-4, IL-13 and TGF-β cytokines, leading to macrophage activation and EMT in epithelial cells.

TLRs, especially TLR2, TLR4 and TLR9, play a central role in linking innate and acquired immunity. HDM-sensitized individuals show increased expression of TLR2 and TLR4 in the nasal epithelium, which correlates with the severity of clinical symptoms. The activation of TLR4 by cleaved plasma proteins such as fibrinogen may enhance Th2-type responses, and in addition, TLR9 regulates the response through the induction of IL-2 and control of IL-4, IL-5 and IL-13 expression as it is shown on Figure 2 [60,61].

### 5.9. Alternative Pathways for Initiating the Inflammatory Response

Recent reports have shown that certain HDM proteases can initiate the inflammatory response independent of the presence of IgE. This is possible due to the activation of neuronal receptors (e.g., TRPV1) and the secretion of DAMPs, such as ATP or uric acid, which attract antigen-presenting cells and promote the development of a Th2-type response. Importantly, proteases also act as adjuvants, enhancing the immunogenicity of other proteins lacking enzymatic activity [59].

Furthermore, recent evidence highlights a central role for TRPV1—mediated neuroimmune crosstalk in both skin and airway responses to HDM. Serhan et al. demonstrated that HDM cysteine proteases directly activate TRPV1^+^ Tac1^+^ nociceptors, which form sensory clusters with MRGPRB2^+^ mast cells in the skin; the release of substance P from these neurons triggers rapid mast cell degranulation and drives early type 2 inflammation in atopic dermatitis models, identifying TRPV1 as a key initiator of cutaneous allergic responses [62].

Manti et al. reviewed analogous mechanisms in the respiratory tract, showing that TRPV1 upregulation contributes to cough hypersensitivity, airway neurogenic inflammation and bronchoconstriction; they further describe a bidirectional interplay between TRPV1 and β_2_-adrenergic receptors, whereby defective β_2_AR signaling (e.g., during viral infections or prolonged β-agonist use) exacerbates TRPV1-driven neuroinflammation and worsens asthma control [63].

These data indicate that TRPV1 antagonists or modulators of the TRPV1–β_2_AR axis may represent promising strategies to dampen both skin and airway allergic inflammation induced by HDM.

### 5.10. Therapeutic Implications and Prospects

Understanding the molecular mechanisms underlying HDM allergy opens up new therapeutic opportunities. Promising strategies include

- Inhibitors of TLR pathways (e.g., TLR4);
- Neutralization of IL-33, TSLP and PAR2;
- Use of mutant allergens lacking enzymatic activity in immunotherapy;
- Drugs modulating the activity of sensory neuron receptors (TRPs);
- Selective protease inhibitors (ADIs—allergen delivery inhibitors);
- Selective inhibition of MFI or its receptor;
- Modulation of innate immune training (e.g., by methyltransferase inhibitors);
- Blocking EMT (e.g., by inhibiting TGF-β1, the Wnt pathway);
- Targeting subtypes of immune cells that overreact to HDM.

A complete understanding of the interplay between proteolytic allergen activity, innate immune receptors and neuronal pathways that initiate allergy is still lacking. This points to the need to intensify research on precise mapping of molecular interactions in the first phases of the allergic response. The mechanisms that differentiate the response according to the specific lipid composition of allergens and how metabolic changes fix the phenotype of immune cells in a proallergic direction remain unexplained [56,57,60,61].

## 6. New Developments in Allergen-Specific Immunotherapy

Allergen-specific immunotherapy (AIT) is the only allergy treatment available that not only relieves the symptoms of the disease but also modifies the underlying immune mechanisms. In recent years, intensive research on AIT in the context of HDM has focused on evaluating the efficacy of therapy, identifying predictive biomarkers and developing new optimization strategies.

One of the key factors influencing the effectiveness of AIT is the quality of the allergen extracts used. It has been shown that allergen preparations, both diagnostic and therapeutic, should, to the greatest extent possible, reflect the full spectrum of major, midtier and minor allergens naturally present in the allergen source. Meanwhile, commercially available formulations are often characterized by variable molecular composition, and incomplete representation of allergens can lead to limited therapeutic efficacy.

Particular attention has been paid to the allergen Der p 23, which is now recognized as a major allergen of *Dermatophagoides pteronyssinus*. Its presence—and, in many cases, absence or insufficient amount—in mite extracts is important for the effectiveness of AIT. Studies indicate that in patients sensitized to Der p 23, the efficacy of immunotherapy may be reduced, likely due to the lack of standardization of this allergen in available vaccines. Therefore, precise evaluation and control of Der p 1, Der p 2 and Der p 23 content in allergen preparations is becoming a key element of quality assurance. The development of high-resolution mass spectrometry (LC-MS/MS) technology has significantly improved the ability to assess the molecular composition of extracts, setting new standards for quality control and consistency between batches [64,65,66].

Consequently, contemporary efforts to improve allergen immunotherapy include not only the individualization of therapy and the development of new formulations but also ensuring that relevant allergens are fully represented in therapeutic preparations, which is critical to maximizing treatment efficacy and clinical benefits for patients.

Additionally, given the pivotal role of accurate diagnosis in guiding AIT, in vitro assays have seen significant advances. Component-resolved diagnostics (CRD) platforms, such as ImmunoCAP and microarray-based assays (e.g., ImmunoCAP ISAC), now quantify specific IgE against individual HDM molecules (Der p 1, Der p 2 and Der p 23, as well as midtier allergens like Der p 5 and Der p 7). These assays demonstrate high sensitivity (up to 90%) and specificity (approximately 85%) for detecting clinically relevant sensitizations, and their results correlate strongly with symptom severity and challenge test outcomes. Moreover, basophil activation tests (BATs) using flow cytometry offer functional insight into allergen reactivity, with reported sensitivities of 75–80% and specificities exceeding 90% for HDM allergens. Incorporating these advanced diagnostics into the clinical workflow enhances patient stratification, predicts AIT responsiveness more accurately and ultimately informs personalized treatment strategies [67,68,69].

### 6.1. Biomarkers of AIT Efficacy

One of the key areas of AIT research is the identification of biomarkers to predict patient response to therapy. Recent studies have shown that the efficacy of subcutaneous immunotherapy (SCIT) can be predicted by the ratio of specific IgE to total IgE (sIgE/tIgE), the ratio of follicular regulatory cells (TFR) to type 2 follicular helper T cells (TFH2) and the incidence of switched memory B cells (BSM). A predictive model based on these biomarkers has shown high performance in predicting treatment response, which may enable a more personalized approach to therapy [70,71,72,73,74].

In addition, a retrospective study of patients with asthma and/or allergic rhinitis showed that early improvement of symptoms in the first phase of therapy may indicate its long-term effectiveness. They found that patients whose symptom and medication use score (edSMS) fell by at least 18.4% after the first phase of therapy were more likely to have an effective response to treatment after three years [75,76].

### 6.2. Neosensitization During AIT

Neosensitization, or the development of new sensitization to vaccine components, can occur during immunotherapy. Studies have shown that about 69% of patients undergoing immunotherapy with HDM extracts acquire IgE positivity to the Der p 3 component after one year of therapy. However, levels of specific IgG4 and subjective symptom improvement (VAS) were not significantly different between patients who experienced neosensitization and those who did not. Further studies on the clinical significance of this phenomenon are needed [77,78,79,80,81].

### 6.3. Immunomodulation and Metabolism During AIT

Studies have shown that AIT not only affects the immune system but can also modulate metabolism, especially with regard to fatty acids. After 12 months of SCIT therapy, significant changes were observed in the levels of polyenoic fatty acids, such as arachidonic acid, docosahexaenoic acid (DHA) and eicosapentaenoic acid (EPA). Interestingly, these changes correlated with levels of mite allergen-specific IgG4, suggesting a possible role for lipid metabolism in the mechanisms of immunotherapy [82,83,84,85,86].

### 6.4. The Role of Basophils and ILC Cells in the Mechanisms of AIT

Studies on the mechanisms of AIT indicate an important role for basophils in regulating the immune response. In patients undergoing SCIT with HDM allergens, a significant reduction in basophil activity against inhaled allergens was observed after therapy, which may be related to the inhibition of Th2 cell activation. RNA sequencing from isolated basophils showed reduced expression of genes responsible for immune cell activation, antigen presentation and Th2 cell differentiation, which may contribute to the long-term efficacy of the therapy [87,88,89,90,91,92].

New reports also suggest that innate lymphoid cells (ILCs), particularly the ILC2 subtype, may play a key role in the mechanisms of AIT. A subset of IL-10-producing ILC2 has been identified that is associated with immune tolerance after pollen immunotherapy and mite allergy. Their presence may be an important indicator of therapy efficacy [93,94,95].

### 6.5. Safety and New Approaches in Immunotherapy

A novel approach in immunotherapy is intratonsillar immunotherapy (ITIT), the administration of allergens by injection into the tonsils. Studies have shown that this method can effectively relieve symptoms of allergic rhinitis in the short term, but its long-term efficacy remains uncertain. ITIT led to an increase in specific IgG4 antibodies, although to a lesser extent than SCIT. The therapy was well tolerated, and side effects were mainly limited to transient sore throat. Further research is needed to optimize dosing and evaluate the long-term benefits of this method [96,97,98,99].

In contrast, oral immunotherapy (OIT) with enteral capsules showed greater efficacy in reducing symptoms of allergic rhinitis than SCIT, which may open up new therapeutic possibilities [100,101].

### 6.6. Combination Therapy of SCIT and Dupilumab

New research suggests that combining SCIT with dupilumab for up to 144 weeks significantly improves outcomes for patients with severe atopic dermatitis (AD) and induces beneficial changes in serum biomarkers associated with AIT. Combination therapy led to a significant reduction in levels of total IgE and specific IgE against *D. pteronyssinus* and *D. farinae* compared to SCIT alone. A significant increase in sIgG4 was observed in the SCIT and SCIT/dupilumab groups, while dupilumab monotherapy showed no such effect. The combination of SCIT and dupilumab may have long-term benefits, including reduced dependence on biologic therapy and sustained remission of symptoms [102,103,104,105,106].

## 7. Materials and Methods

This publication is a review and was based on an analysis of recent scientific reports on HDM allergy. A literature search was conducted in the PubMed, Scopus and Web of Science databases, focusing on publications from recent years to include the most current information. The search was based on a combination of keywords, such as house dust mite allergy, Der p 1, Der p 2, allergen-specific immunotherapy, biomarkers of AIT efficacy and recombinant allergens, to select relevant literature items.

To ensure the reliability of the review, the analysis primarily included articles from peer-reviewed journals with a high impact factor, meta-analyses and systematic reviews. Experimental and clinical papers were analyzed in terms of methodology and the size of the study population, which allowed for the selection of studies with the highest quality of evidence. Works of limited merit were excluded, including reports with small study samples, most case studies and articles with limited data. Particular attention was paid to new immunotherapy strategies and potential biomarkers of AIT efficacy, allowing for the presentation of current research directions in the field.

The collected data were critically analyzed by the authors to draw conclusions about the epidemiology, pathophysiology and advances in the diagnosis and treatment of house dust mite allergy.

## 8. Conclusions

House dust mite allergy is one of the most common allergic diseases, and its diagnosis, despite the availability of skin testing and specific IgE determination, still faces difficulties in accurately determining the sensitization profile. Key allergens, such as Der p 1, Der p 2 and Der p 23, play an important role in sensitization mechanisms, and some, such as Der p 10, can cause cross-reactions. It is noteworthy that midtier and minor allergens, especially Der p 21, are also becoming increasingly important, although further research is needed on their role in disease development and response to immunotherapy.

This review highlights that midtier and minor allergens—often overlooked in standard diagnostic panels—may significantly influence the clinical picture and therapy outcomes. The inclusion of such components in diagnostic testing and immunotherapy formulations should be considered essential for personalized treatment approaches.

Allergen-specific immunotherapy (SCIT and SLIT) remains the only disease-modifying method, although standard allergen extracts are not always optimal. New approaches, such as immunotherapy with recombinant allergens or combination therapies (e.g., with dupilumab), may improve treatment efficacy. Studies of biomarkers, such as the sIgE/tIgE ratio or basophil activity, indicate their potential in predicting response to therapy. In addition, immunotherapy affects lipid metabolism, which may be relevant to its efficacy. Promising results are also being obtained in new treatments, such as immunotherapy of the palatine tonsils (ITIT), oral immunotherapy (OIT) and enteral capsule administration.

The role of immune cell metabolism and epithelial remodeling is increasingly recognized as central in HDM-induced allergy. Molecular mechanisms involving TRPC1 calcium channels, macrophage polarization and epithelial–mesenchymal transition (EMT) open new therapeutic possibilities by targeting structural cells and innate immune responses, rather than just IgE-mediated pathways.

Moreover, the integration of advanced in vitro diagnostics—particularly component-resolved diagnostics (CRD) for specific IgE to individual HDM molecules and basophil activation tests (BATs)—provides high sensitivity and specificity in detecting clinically relevant sensitizations, with strong correlations to symptom severity and challenge test outcomes. Incorporating these assays into routine clinical practice enhances patient stratification, guides personalized AIT regimens, and enables more accurate monitoring of treatment response.

Recent evidence emphasizes that optimal clinical outcomes depend on a precise match between the patient’s molecular sensitization profile and the composition of therapeutic extracts—especially regarding the presence of Der p 23. The use of mass spectrometry and molecular profiling is critical in ensuring extract quality and consistency.

The future of treatment for house dust mite allergy depends on further personalization of therapy, the development of precise diagnostic methods and a better understanding of the immunological and metabolic mechanisms involved in the immunotherapy process. In particu63lar, future studies should aim to standardize and validate these in vitro diagnostic tools across diverse patient populations, to fully harness their potential in optimizing therapeutic decision-making and improving long-term outcomes.

## Figures and Tables

**Table 1:** Taxonomic classification of house dust mites with characteristics of clinically significant species.

Taxonomic Level	Taxon	Description
Cluster	Arachnida (arachnids)	Arachnids are a cluster of arthropods that includes organisms such as spiders, scorpions and mites. They are characterized by the presence of four pairs of legs, the absence of feelers and the division of the body into two main parts: the cephalothorax and the abdomen.
Order	Acariformes (proper mites)	Proper mites are one of the main orders of mites, encompassing many species with diverse ecology, including saprophytes, parasitoids and predators.
Family	Pyroglyphidae	The Pyroglyphidae family includes mites commonly found in the domestic environment, especially in dust. They are the main allergens responsible for allergic reactions in humans. Species: *Dermatophagoides pteronyssinus*: The most common species of house dust mite in Europe, including Poland. It prefers relative humidity above 70% and temperatures around 25 °C. It feeds mainly on exfoliated human and animal skin. *Dermatophagoides farinae*: It is common across North America and also identified in temperate/subtropical regions of Europe, Asia and Australia. It has similar environmental requirements to *D. pteronyssinus*, but it tolerates slightly lower humidity. *Euroglyphus maynei*: A less common species in domestic environments; it may contribute to allergies in susceptible individuals.
Family	Glycyphagidae	Mites of this family are often found in food warehouses but can also be found in homes, especially where organic products are stored. Species: *Lepidoglyphus destructor*: It is known as a storage pest but can also occur in homes storing grain products. *Glycyphagus domesticus*: It is often found in damp home areas (basements, pantries); it can cause allergic reactions in sensitive individuals.
Family	Acaridae	Mites in this family are commonly known as pests of food products, but they can also be found in house dust. Species: *Acarus siro*: Known as the flour mite, it often infests grain products but also occurs in house dust. *Tyrophagus putrescentiae*: It is found in high-protein foods (cheese, nuts) and moist home areas.
Family	Echimyopodidae	Mites in this family are less well known but can occur in the home environment and affect human health. Species: *Blomia tropicalis*: It is common in tropical and subtropical regions but can also occur in other climate zones; it is considered an important allergen in some countries.

**Table 2:** List of selected house dust mite allergens with their characteristic features [31,32,33,34,35,36,37,38,39,40,41,42,43,44,45,46,47,48,49,50,51,52,53].

Allergen Group No.	Abbreviation by Species		IgE Reactivity	Biological and Clinical Significance
	*D. pteronyssinus*	*D. farinae*		
1	Der p 1	Der f 1	90%	These are proteolytic enzymes belonging to the cysteine protease group. Their action degrades intercellular junctions in the airway epithelium, which facilitates the penetration of allergens and the initiation of the immune response. Significantly higher levels are observed in children with asthma than in children without asthma.
2	Der p 2	Der f 2	90%	These proteins act as modulators of the immune response. They bind lipids to the ML domain. Structurally, they resemble TLR4 receptors, thus stimulating innate response mechanisms and enhancing the allergic response. Significantly higher levels are observed in children with asthma than in children without asthma.
3	Der p 3	Der f 3	55%	These are proteolytic enzymes with trypsin-like function. They have the ability to break down epithelial cell membranes, which increases the permeability of other allergens.
4	Der p 4	Der f 4	15%	This is an alpha-amylase that can cross-react with other food allergens, such as those of plant origin. It causes allergic reactions in patients allergic to mites. High titers of IgE-binding anti-alpha amylase antibodies are observed in patients infected with scabies or reporting previous exposure to scabies.
5	Der p 5	Der f 5	35%	These are proteins of unknown function, but they have been shown to strongly stimulate the immune system, leading to the production of IgE antibodies. Significantly higher levels are observed in children with asthma than in children without asthma.
6	Der p 6	Der f 6	53%	Chymotrypsin is a proteolytic enzyme that can destroy the epithelial barrier in the respiratory tract.
7	Der p 7	Der f 7	40%	These are proteins with a structure that resembles bacterial membranes, making their mechanism of action likely to resemble endotoxin activity.
10	Der p 10	Der f 10	42%	Tropomyosin is a protein with high homology to seafood tropomyosins, which means it can cause cross-reactions in patients allergic to shellfish.
11	Der p 11	Der f 11	45%	Paramyosin is a muscle protein of mites that shows the ability to stimulate the immune system. It is used clinically as a serological marker for HDM-associated atopic dermatitis.
14	Der p 14	Der f 14	43%	Apolipoprotein, a protein with a potential role in the immune response.
15	Der p 15	Der f 15	43%	Chitinase, a chitin-degrading enzyme that may play a role in allergic reactions.
20	Der p 20	Der f 20	25%	Arginine kinase, potentially associated with allergic reactions in people with asthma. It has been clinically correlated with HDM-related asthma, as well as active scabies infection.
23	Der p 23	Der f 23	77%	Peritrophin-like protein plays a key role in stabilizing the intestinal barrier of mites. Significantly higher levels are observed in children with asthma than in children without asthma.

## Data Availability

The original contributions presented in this study are included in the article. Further inquiries can be directed to the corresponding author.

## Abbreviations

The following abbreviations are used in this manuscript:

HDM	House dust mites
AIT	Allergen-specific immunotherapy
OIT	Oral immunotherapy
SCIT	Subcutaneous immunotherapy
SLIT	Sublingual immunotherapy
ITIT	Intratonsillar immunotherapy
BMDM	Bone marrow-derived macrophages
BMDAM	Bone marrow-derived alveolar macrophages
BMDC	Bone marrow-derived dendritic cells
TRPC1	Transient receptor potential canonical 1
EMT	Epithelial–mesenchymal transition
TJs	Tight junctions
AD	Atopic dermatitis
